# Assembly and Annotation of the Complete Genome Sequence of T4-Like Bacteriophage 132

**DOI:** 10.1128/MRA.00649-21

**Published:** 2021-09-30

**Authors:** C. Roush, B. K. Chan, P. E. Turner, A. R. Burmeister

**Affiliations:** a Department of Ecology and Evolutionary Biology, Yale University, New Haven, Connecticut, USA; b BEACON Center for the Study of Evolution in Action, East Lansing, Michigan, USA; c Program in Microbiology, Yale School of Medicine, New Haven, Connecticut, USA; Queens College CUNY

## Abstract

Here, we describe the complete sequence of bacteriophage 132, a T4-like Escherichia coli phage. Phage 132 has a genome of 166,922-bp length, with 286 predicted genes.

## ANNOUNCEMENT

Bacteriophage 132 was isolated from feces from a swine farm in Connecticut, USA. Like phage T4, it is reliant on host lipopolysaccharide (LPS) synthesis genes (1); however, its outer membrane protein receptor is likely OmpF (1), rather than OmpC (the T4 receptor) (2).

Swine feces were resuspended in phosphate-buffered saline with 10 mM MgSO_4_. This mixture was centrifuged at 3,220 × *g* for 15 min and filtered through a 0.22-μm filter. The filtrate was enriched in tryptic soy broth and Escherichia coli strain BW25113 for 24 h. Plaques were triple isolated and amplified to high titer. DNA was extracted using the Norgen Biotek phage DNA isolation kit. The sequencing library was prepared using the Nextera DNA Flex library preparation kit and sequenced at Felix Biotechnology on an Illumina NextSeq system, yielding 150-bp paired-end reads. Only the R1 reads were used for annotation. Genome annotation and assembly were conducted in the Galaxy (3) and Web Apollo (4) phage annotation platforms. Default parameters were used for all software unless otherwise noted. Sequencing reads were rarified to 250× coverage to improve assembly using FastQ Subset v.1.1 (5, 6) and trimmed using the Trim Sequences tool v.1.0.2 (7). Reads were quality checked using FastQC v.0.72 (8), and a contig was assembled at 128× coverage using SPAdes v.3.12.0 (9). One other contig, a 56-bp poly(G) artifact from sequencing (10), was omitted from further analysis. One of the overlapping ends (55 bp), which indicated a circular genome, was trimmed using the Trim Sequences tool v.1.0.2 (7).

Using BLASTn (11) with the complete genome, phage 132 was found to be closely related to the well-characterized phage T4 (GenBank accession number MT984581), with 95.36% nucleotide identity over 88% of the phage 132 genome. The complete assembled contig was reopened in alignment with the T4 reference genome using Reopen Fasta Sequences v.2.0 (5, 6). This sequence was imported into Apollo using the Galaxy Structural Phage Annotation Workflow v.2020.07 (6). Gene locations were predicted by weighing evidence from the algorithms GLIMMER3 v.0.2 (12), MetaGeneAnnotator v.1.0.0 (13), and SixPack v.5.0.0 (14). Final protein-coding gene calls were made manually, based on the presence of a valid start codon, the presence of a valid Shine-Dalgarno sequence, and assessment of gaps between genes. Calls for tRNAs were made by examining evidence from tRNAScan-SE v.0.4 (15) and ARAGORN v.0.6 (16). One predicted gene spanned the ends of the genome; therefore, we adjusted the genome start location using Reopen Fasta Sequences v.2.0 (5, 6) to keep the ends of that gene together. Functional predictions were made manually using the Galaxy Functional Phage Annotation Workflow v.2020.09 (6) and comparing BLASTp (17, 18) results from three phage genome databases (the canonical phages, nonredundant-all phages, and Swiss-Prot databases). The geecee tool v.5.0.0 (19) was used to determine that the GC content of this genome is 35%.

The phage 132 genome contains 286 predicted genes, 150 of which are annotated as hypothetical proteins. A total of 126 predicted genes are annotated as putative proteins with predicted functions, and 10 tRNA genes are identified.

### Data availability.

The annotated phage 132 genome is available in GenBank under accession number MZ417521. The rarified sequence reads are available in the Sequence Read Archive (SRA) with SRA accession number SRP325389. The BioProject accession number is PRJNA740247.
